# Supplementary material for: Convergent and Divergent Mitochondrial Pathways as Causal Drivers and Therapeutic Targets in Neurological Disorders
Source: Curr Issues Mol Biol. 2025 Aug 8;47(8):636. doi: 10.3390/cimb47080636 (PMC12384102; doi:10.3390/cimb47080636)
Supplement: Supplementary file 1 [file cimb-47-00636-s001.zip › cimb-3742969-SupplementaryFigures-R1-proof.pdf]

Supplementary Figure

Supplementary Figure S1. Performance Distributions of Machine Learning Algorithms Across Neurological Diseases........2
Supplementary Figure S2. A Multi-Faceted Performance Analysis of Predictive Models for Four Neurological Diseases. 4
Supplementary Figure S3. Pathway Performance Analysis and Evaluation Across Neurological Diseases........6
Supplementary Figure S4. Mendelian Randomization Analysis of Top 5 Genes in Alzheimer's Disease........8
Supplementary Figure S5. Mendelian Randomization Analysis of Top 5 Genes in Amyotrophic Lateral Sclerosis 9
Supplementary Figure S6. Mendelian Randomization Analysis of Top 5 Genes in Multiple Sclerosis 10
Supplementary Figure S7. Mendelian Randomization Analysis of Top 5 Genes in Parkinson's Disease........11
Supplementary Figure S8. Colocalization Analysis of eQTLs and Disease Risk Variants 12

.

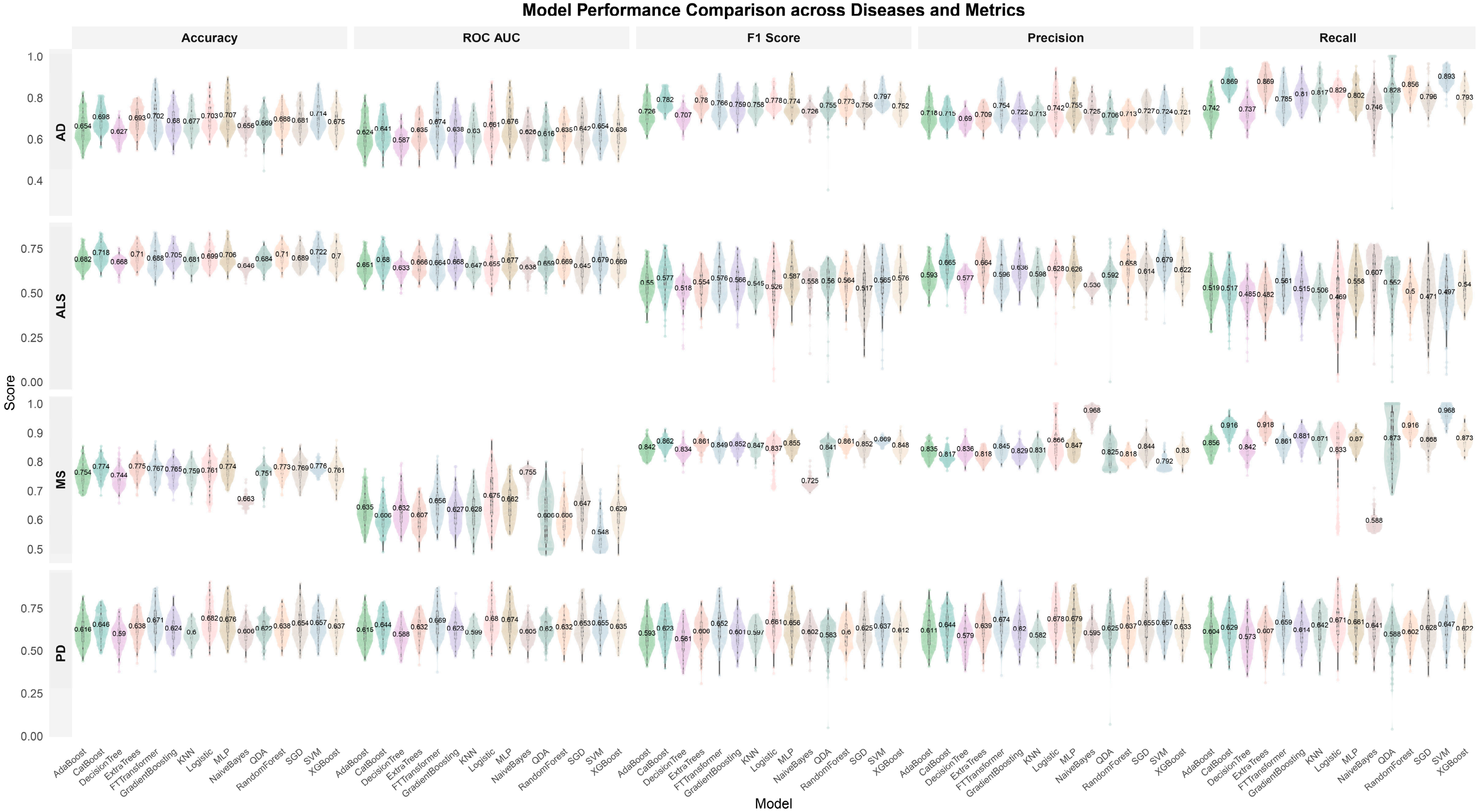

Supplementary Figure S1. Performance Distributions of Machine Learning Algorithms Across Neurological Diseases

The performance distributions of fifteen distinct machine learning algorithms are visualized as violin plots, organized by disease type and across five evaluation metrics: Accuracy, ROC AUC, F1 Score, Precision, and Recall. Each plot displays the density of performance scores from cross-validation runs, with the black dot marking the median value. Notable findings include the superior and consistent performance of Support Vector Machines (SVM) and Multi-layer Perceptron (MLP), with SVM achieving the highest Accuracy in MS (0.776) and a top Recall (0.968). On the

other hand, simpler models like DecisionTree consistently underperformed, particularly in PD (Accuracy: 0.590), highlighting the model's limitations for this complex dataset. Ensemble methods like CatBoost and ExtraTrees also demonstrated robust and competitive performance across various conditions.

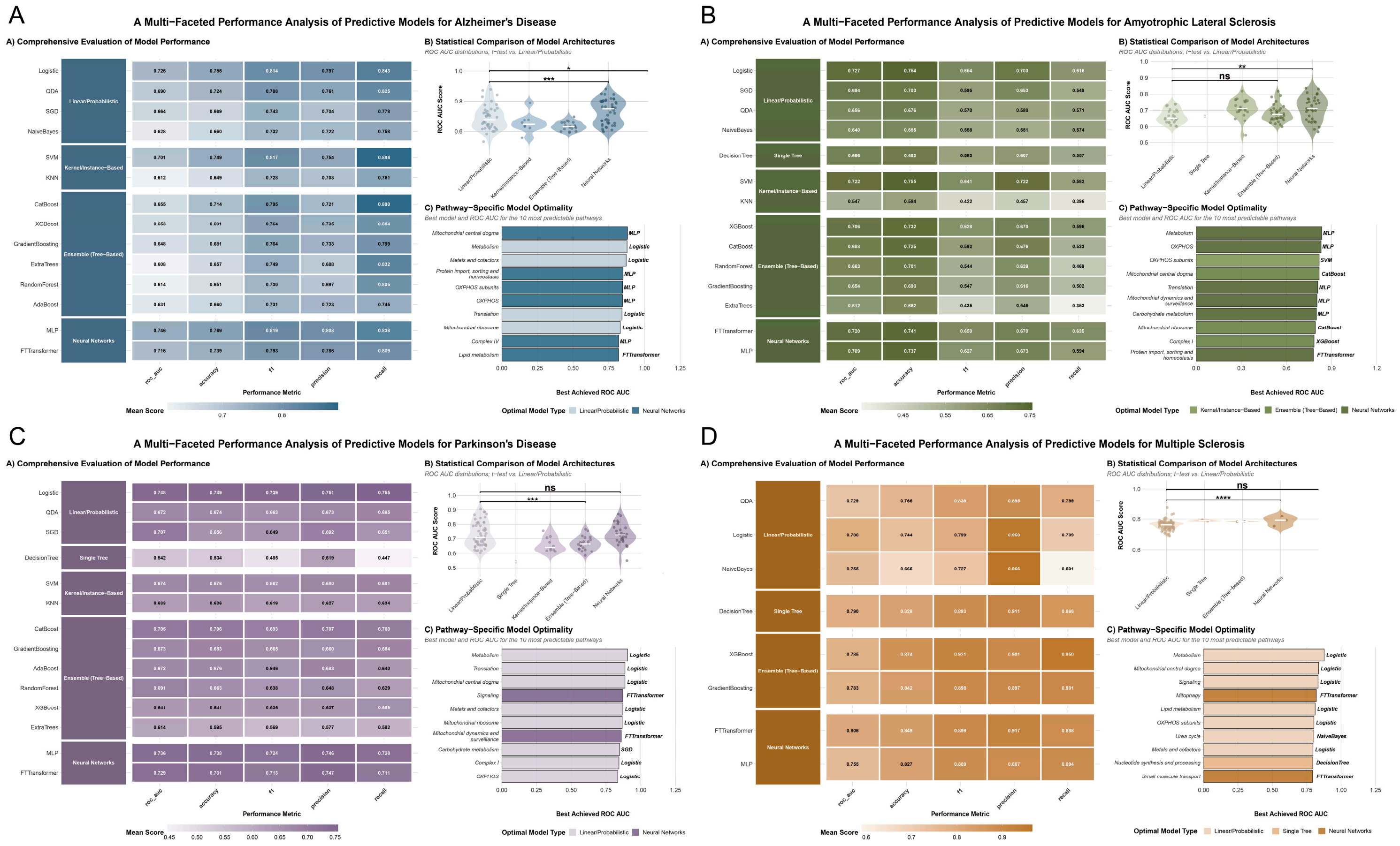

Supplementary Figure S2. A Multi-Faceted Performance Analysis of Predictive Models for Four Neurological Diseases. The figure presents a comprehensive comparison of 15 machine learning models, grouped by architectural type, for the classification of (A) Alzheimer's Disease (AD), (B) Amyotrophic Lateral Sclerosis (ALS), (C) Parkinson's Disease (PD), and (D) Multiple Sclerosis (MS) versus

healthy controls. Each panel contains three components:

(A) Comprehensive Evaluation of Model Performance: A heatmap displaying the mean performance of each model across five metrics. Models are ranked by their overall mean score. For example, in AD, Neural Networks (MLP, FTTransformer) and Logistic Regression achieved the highest overall scores, while in MS, Ensemble models like XGBoost were top performers.

(B) Statistical Comparison of Model Architectures: Violin plots comparing the ROC AUC distributions of different model architectures, where each point represents the performance on a specific mitochondrial pathway. Statistical significance was determined by a t-test against the Linear/Probabilistic baseline. For AD, both Ensemble ($p < 0.001$) and Neural Network ($p < 0.05$) models showed significant improvement. For ALS, Neural Networks were significantly better ($p < 0.01$). For PD, only Ensemble models showed a significant advantage ($p < 0.001$), while for MS, Ensemble models were also significantly superior ($p < 0.0001$).

(C) Pathway-Specific Model Optimality: A bar chart illustrating the best-achieved ROC AUC and the corresponding optimal model for the 10 most predictable mitochondrial pathways. The color of the bars indicates the architectural type of the optimal model. This reveals that signal complexity varies by pathway; for instance, in AD, the “Mitochondrial central dogma” pathway was best modeled by a Neural Network (MLP, ROC AUC = 0.88), whereas the "Metabolism" pathway was optimally modeled by a simpler Logistic Regression (ROC AUC = 0.88).

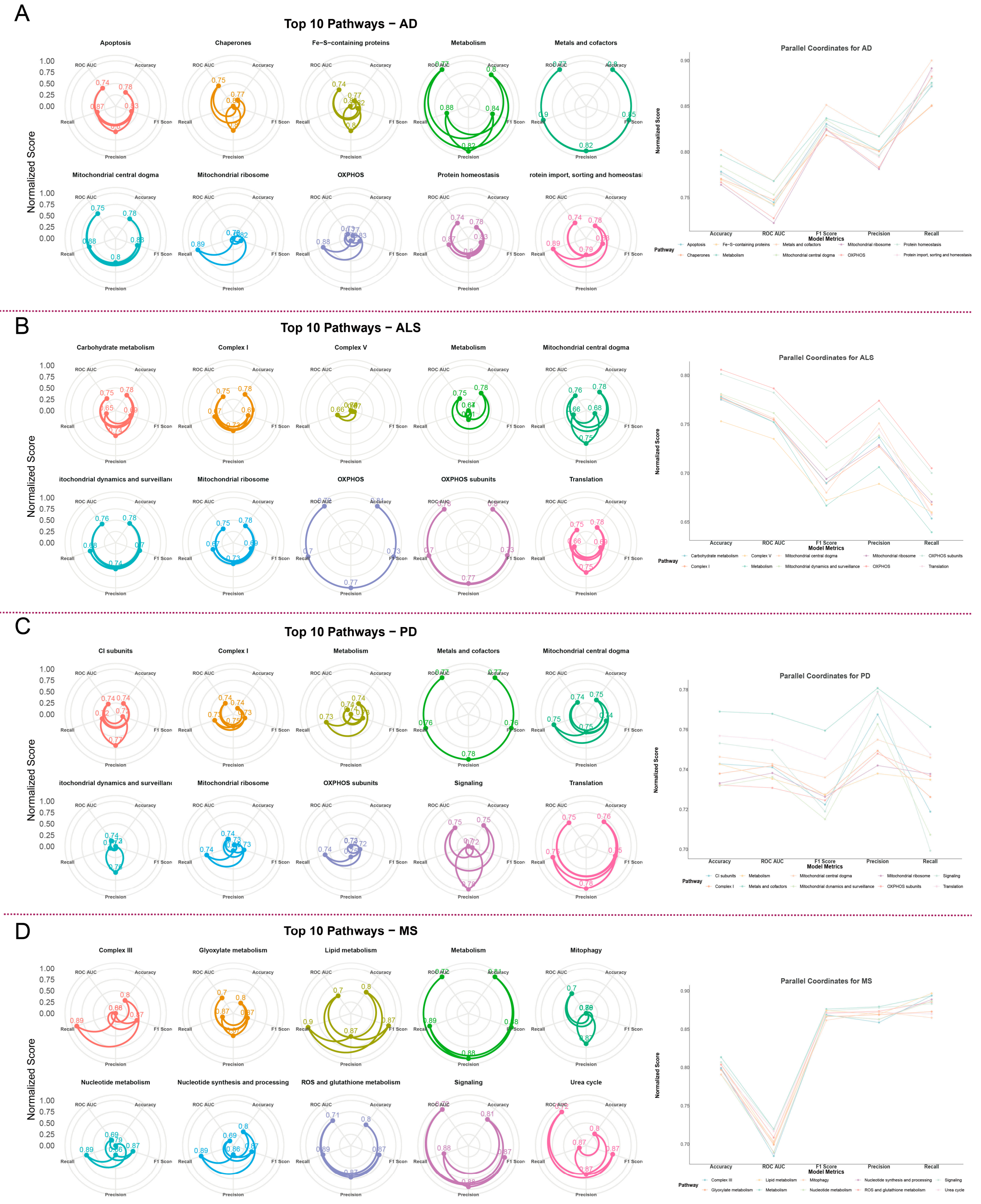

Supplementary Figure S3. Pathway Performance Analysis and Evaluation Across Neurological Diseases

This figure illustrates the performance of the top 10 mitochondrial pathways associated with four neurological diseases: Alzheimer's Disease (AD), Amyotrophic Lateral Sclerosis (ALS), Parkinson's Disease (PD), and Multiple Sclerosis (MS), evaluated through multiple performance metrics (Accuracy, ROC AUC, Precision, Recall, and F1 Score).

(A) Top 10 Pathways for AD: Radar plots display the performance of key mitochondrial pathways in AD, with pathways like Apoptosis and Chaperones showing high normalized scores across various metrics. The parallel coordinates plot on the right

further reveals the relative performance of each pathway, highlighting the top-performing features.

(B) Top 10 Pathways for ALS: Radar plots for ALS highlight pathways such as Carbohydrate metabolism and Complex IV, which are critical for ALS pathogenesis, demonstrating strong performance across multiple metrics. The parallel coordinates plot complements the radar plots by showing trends in model performance across different pathways.

(C) Top 10 Pathways for PD: Radar plots for PD show the performance of pathways such as Complex I and Mitochondrial control of dogma, indicating their significant contribution to disease classification. The parallel coordinates plot helps to visualize the differences in pathway performance, particularly for metrics like ROC AUC.

(D) Top 10 Pathways for MS: Radar plots for MS feature pathways including Complex III and Glycolytic metabolism, providing insights into their relevance for disease prediction. The parallel coordinates plot summarizes the comparative performance of these pathways across different models and metrics.

These analyses provide a comprehensive view of how mitochondrial pathways contribute to the prediction and understanding of neurological diseases, showcasing their varying importance across the four conditions

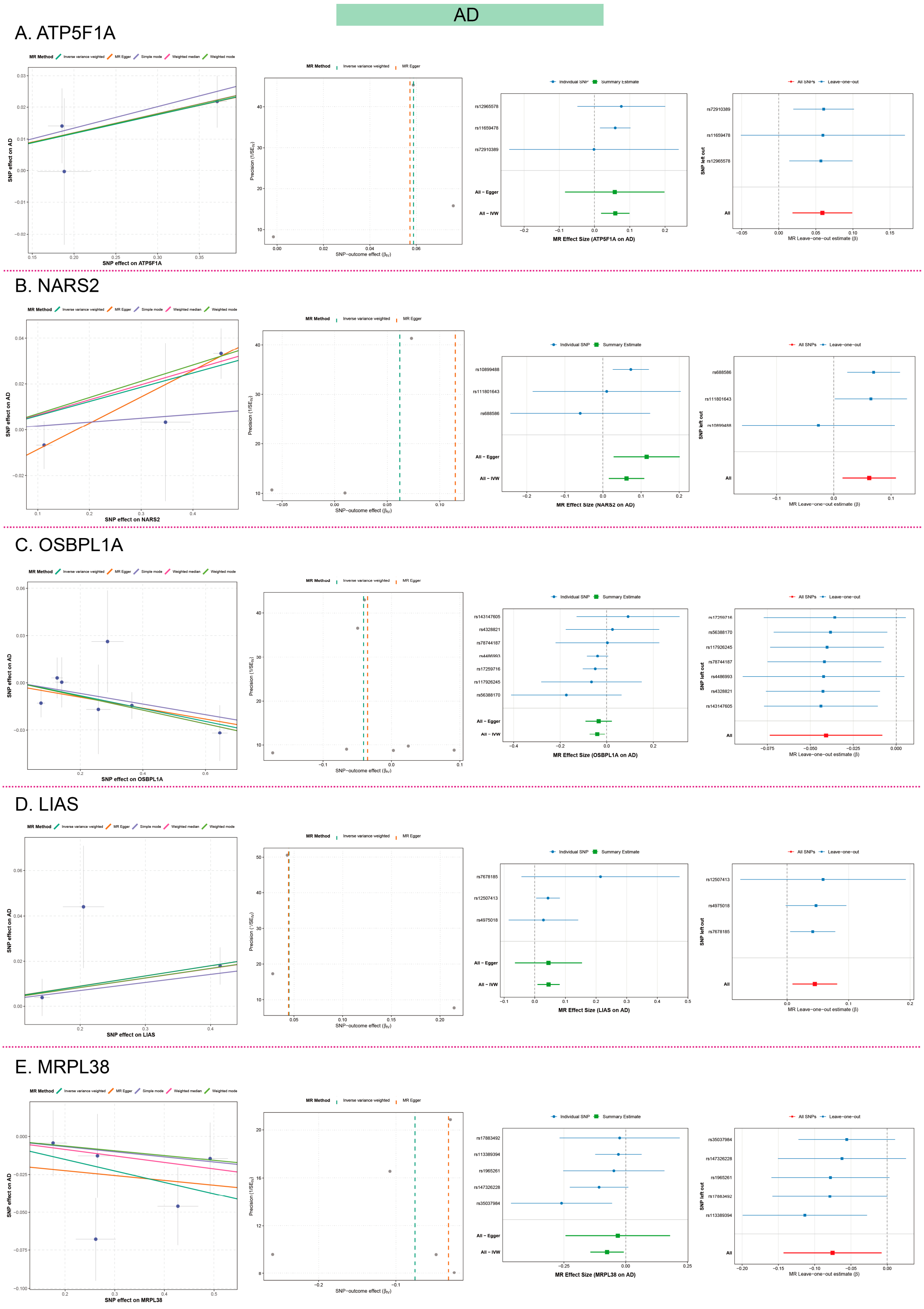

Supplementary Figure S4. Mendelian Randomization Analysis of Top 5 Genes in Alzheimer's Disease

This figure displays MR analysis for the top 5 genes associated with Alzheimer's Disease (AD), highlighting the effect sizes, confidence intervals, and the results of the MR methods (IVW, MR Egger, and others), providing insight into the genetic underpinnings of AD and the significance of mitochondrial pathways.

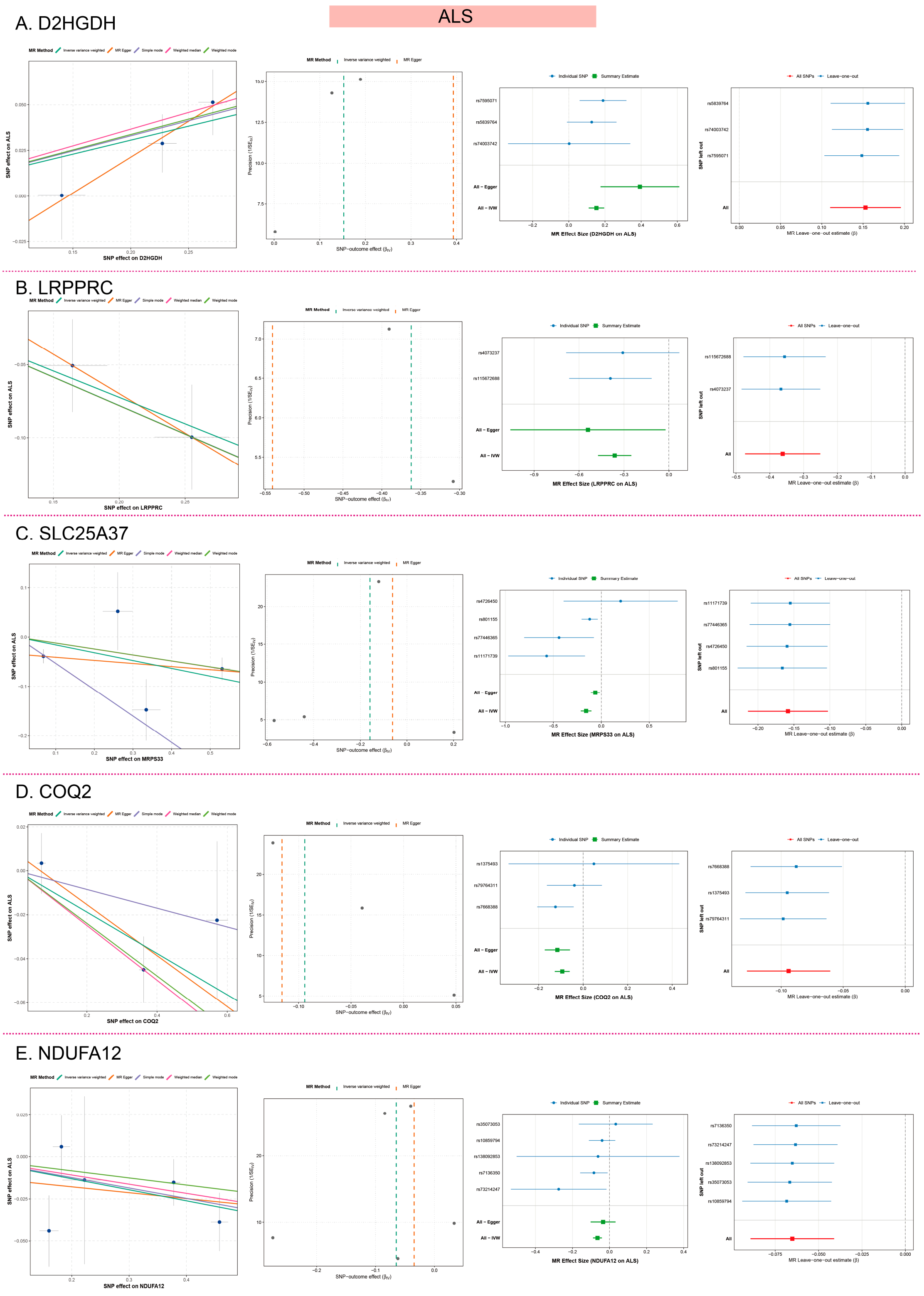

Supplementary Figure S5. Mendelian Randomization Analysis of Top 5 Genes in Amyotrophic Lateral Sclerosis

This figure presents the MR analysis of the top 5 genes implicated in ALS, showcasing the relationship between SNPs, effect sizes, and 95% confidence intervals to explore how mitochondrial-related genes contribute to ALS risk.

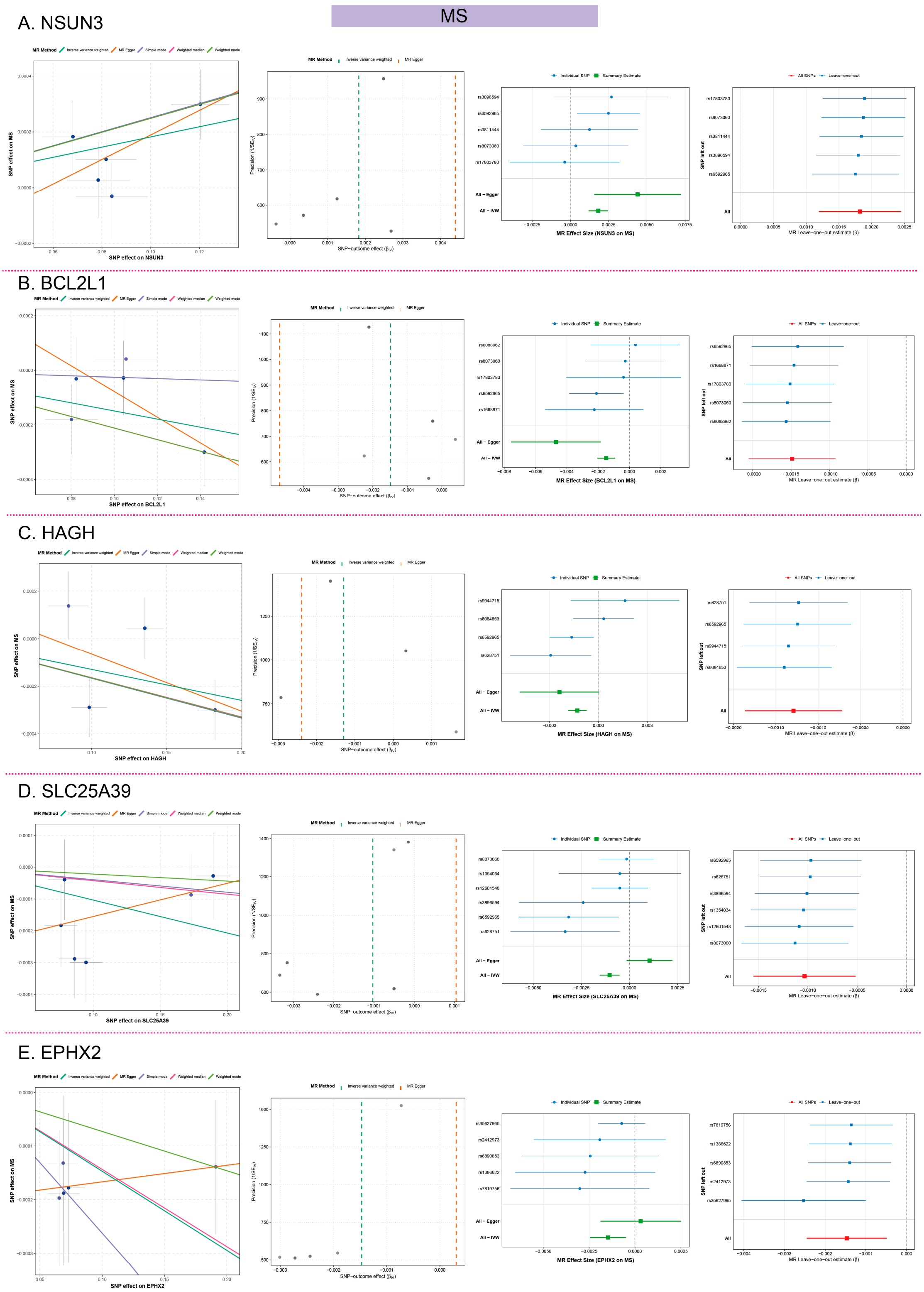

Supplementary Figure S6. Mendelian Randomization Analysis of Top 5 Genes in Multiple Sclerosis

This figure summarizes the MR analysis for the top 5 genes associated with MS, highlighting the genetic contributions of mitochondrial genes to MS susceptibility based on effect sizes and confidence intervals.

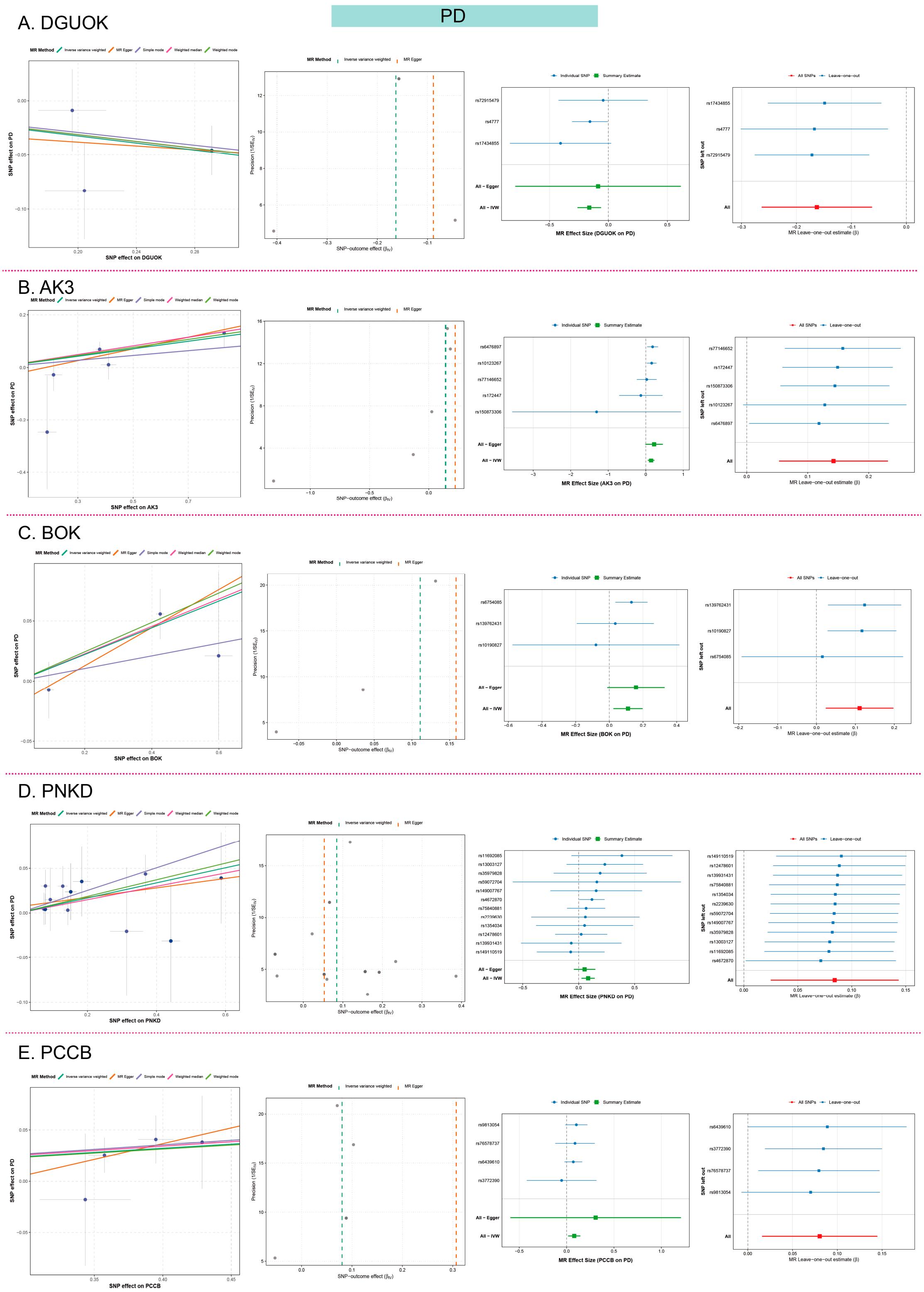

Supplementary Figure S7. Mendelian Randomization Analysis of Top 5 Genes in Parkinson's Disease

This figure shows the MR analysis for the top 5 genes linked to PD, demonstrating the effect sizes and confidence intervals for each gene and their potential role in PD pathogenesis.

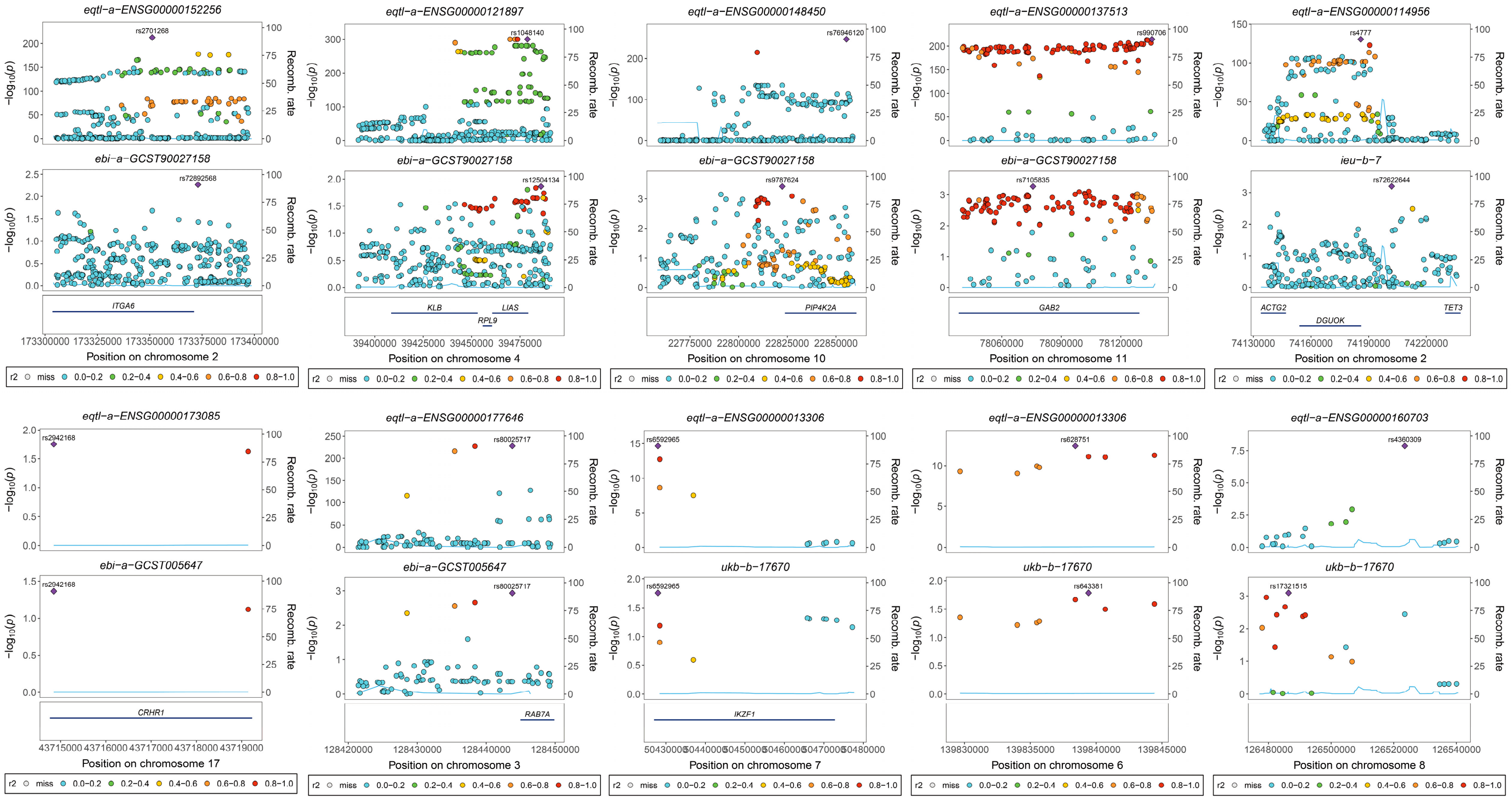

Supplementary Figure S8. Colocalization Analysis of eQTLs and Disease Risk Variants

Colocalization analysis was conducted to assess whether genetic variants influencing mitochondrial pathway-related gene expression (eQTLs) overlap with variants affecting disease risk, providing evidence for shared genetic mechanisms across neurological disorders.
